# Therapeutic efficacy and safety of injectable platelet-rich plasma in women with stress urinary incontinence: a systematic review and meta-analysis

**DOI:** 10.3389/fmed.2026.1728478

**Published:** 2026-03-18

**Authors:** Bobby Indra Utama, Arif Bima Al Birru, Kevin Nathaniel Cuandra, Ratna Puspita, Steven Arianto, Galih Yudo Pranowo, Qanita Saifana, Made Aditya Krisnanta Gandhy, Karisya Juwita Puteri, Rafhy Mifindra, Gabriela Valencia Putri Husodho, Rio Fernando Alexander, Meda Yuliani, Boenga NurCita, Christopher Daniel Tristan

**Affiliations:** 1 Department of Obstetrics and Gynecology, Faculty of Medicine, Universitas Andalas, Padang, Indonesia; 2 Department of Medicine, Faculty of Medicine, Universitas Andalas, Padang, Indonesia; 3 Department of Biochemistry, Faculty of Medicine, Universitas Pembangunan Nasional Veteran Jakarta, Jakarta, Indonesia; 4 Department of Medical Laboratory Technology, Institut Kesehatan Hermina, Jakarta, Indonesia; 5 Department of Medicine, Faculty of Medicine, Universitas Muhammadiyah Yogyakarta, Yogyakarta, Indonesia; 6 Department of Medicine, Faculty of Medicine, Universitas Yarsi, Jakarta, Indonesia; 7 Department of Medicine, Faculty of Medicine, Public Health and Nursing, Universitas Gadjah Mada, Yogya-karta, Indonesia; 8 Department of Medicine, Faculty of Medicine, Universitas Brawijaya, East Java, Indonesia; 9 Department of Medicine, Faculty of Medicine, Universitas Diponegoro, Central Java, Indonesia; 10 Department of Fiscal Administration Science, Faculty of Administrative Sciences, Universitas Indonesia, Jakarta, Indonesia; 11 Department of Midwifery, Faculty of Health Sciences, Universitas Bhakti Kencana Bandung, Bandung, Indonesia; 12 Department of Histology, Faculty of Medicine, Universitas Pembangunan Nasional Veteran Jakarta, Jakarta, Indonesia; 13 Department of Medicine, Faculty of Medicine, Universitas Sebelas Maret, Central Java, Indonesia

**Keywords:** platelet-rich plasma, quality of life, regenerative medicine, stress urinary incontinence, urodynamic outcomes

## Abstract

**Background:**

Stress urinary incontinence (SUI) is a prevalent pelvic floor disorder with significant effects on women’s quality of life. While surgical and non-surgical interventions exist, limitations underscore the need for alternative therapies. Injectable platelet-rich plasma (PRP) has emerged as a regenerative approach targeting tissue restoration. This study aimed to systematically evaluate the efficacy and safety of PRP injections for female SUI.

**Methods:**

This systematic review and meta-analysis adhered to the PRISMA guidelines. Comprehensive searches of PubMed, Scopus, and Cochrane Library up to April 2025 identified randomized controlled trials (RCTs) and quasi-experimental studies assessing PRP for SUI. The review protocol was prospectively registered in the International Prospective Register of Systematic Reviews (CRD420251118485). Eligible studies reported outcomes on symptom severity, urodynamic parameters, quality of life, or adverse events. Risk of bias was assessed using Cochrane RoB 2.0 for RCTs and the NHLBI tool for before–and–after studies. Pooled effect sizes were calculated using random- or fixed-effects models as appropriate.

**Results:**

Eight studies (three RCTs and five quasi-experimental; *n* = 257) were included. PRP significantly improved symptom severity, with pooled reductions in UDI-6 and ICIQ-SF scores at 1–3 months. Urodynamic analysis revealed significant increases in abdominal leak point pressure (MD = 51.07; 95% CI: 36.21–65.93; *p* < 0.0001), while functional profile length remained unchanged. Quality-of-life scores (IIQ-7, ICIQ-SF) showed consistent improvement. Reported adverse events were mild and self-limiting, with no serious complications observed.

**Conclusion:**

Injectable PRP demonstrates promising short-term efficacy in reducing symptoms, improving urodynamic function, and enhancing quality of life in women with SUI, with a favorable safety profile. Larger, standardized RCTs with longer follow-up are needed to validate durability and define its role relative to established therapies.

**Systematic review registration:**

https://www.crd.york.ac.uk/PROSPERO/view/CRD420251118485, CRD420251118485.

## 1 Introduction

Stress urinary incontinence (SUI) is a common pelvic floor disorder affecting 423 million women globally, particularly among those in perimenopausal and postmenopausal age groups (1). SUI has significant implications on women’s quality of life, emotional wellbeing, and daily productivity (2). Despite the availability of several treatment modalities, including pelvic floor muscle training (PFMT), pharmacotherapy, and surgical approaches such as mid-urethral slings, each has its limitations (3). Surgery, while effective, carries risks of complications and may not be acceptable to all patients. Non-surgical treatments, on the other hand, often offer modest results or require sustained adherence for long-term benefit (1, 3).

Against these challenges, regenerative medicine has opened new avenues for managing urogenital dysfunctions. One such innovation is the use of injectable platelet-rich plasma (PRP), an autologous blood derivative rich in growth factors that promote tissue regeneration (4). Injectable PRP, when administered periurethrally or transurethrally, introduces concentrated platelets containing bioactive molecules such as platelet-derived growth factor (PDGF), transforming growth factor-beta (TGF-*β*), and vascular endothelial growth factor (VEGF) (5, 6). These factors are known to stimulate collagen synthesis, angiogenesis, and the recruitment of progenitor cells, mechanisms believed to support the restoration of urethral sphincter function and tissue elasticity in SUI (5, 6).

Preliminary clinical evidence suggests that injectable PRP may improve both subjective symptoms and objective parameters in women with SUI, offering a minimally invasive approach consistent with the principles of personalized and regenerative medicine (6, 7). Unlike conventional bulking agents or pharmacological therapies that primarily provide symptomatic augmentation, PRP is hypothesized to promote tissue regeneration through angiogenic and neurotrophic mechanisms. Although a previous systematic review has summarized available evidence on PRP use in female SUI, quantitative synthesis and focused evaluation of efficacy and safety outcomes specific to SUI remain limited. Therefore, the present study provides an updated systematic review and meta-analysis of randomized and quasi-experimental studies to assess the effects of injectable PRP on symptom severity, urodynamic function, and quality of life, to evaluate adverse events, and to identify gaps to inform future clinical and translational research.

## 2 Methods

### 2.1 Search strategy and selection criteria

This systematic review and meta-analysis were conducted in accordance with the Preferred Reporting Items for Systematic Reviews and Meta-Analyses (PRISMA) guidelines. The review protocol was prospectively registered in the International Prospective Register of Systematic Reviews (CRD420251118485; registered on 8th August 2025). No major deviations from the registered protocol were undertaken. A comprehensive literature search was performed across three major databases: PubMed, Scopus, and the Cochrane Library, to identify relevant clinical studies evaluating the use of injectable platelet-rich plasma (PRP) for the treatment of stress urinary incontinence (SUI) in women. The search spanned from database inception to April 30, 2025, using combinations of the following keywords: (“platelet-rich plasma” OR PRP) AND (“stress urinary incontinence” OR “female stress incontinence” OR “stress incontinence”) AND (injectable OR injection*) AND (women OR female*). Additional citation tracking of included studies was conducted manually to identify further eligible articles.

### 2.2 Eligibility criteria

Studies were eligible for inclusion if they met the following criteria: (1) involved adult female participants with a clinical diagnosis of pure or predominant SUI; (2) evaluated injectable PRP as a primary therapeutic intervention for SUI; (3) reported at least one clinically relevant outcome, including symptom severity scores, urodynamic parameters (e.g., abdominal leak point pressure or functional profile length), health-related quality-of-life measures, or adverse events; and (4) employed a randomized controlled trial (RCT) or quasi-experimental study design. No restrictions were applied regarding PRP preparation protocols, platelet concentration, activation status, injection technique (transvaginal, transurethral, periurethral, or cystourethroscopic), or anatomical injection site; however, such methodological variations were systematically documented and considered sources of clinical heterogeneity. Exclusion criteria comprised studies involving mixed urinary incontinence without predominant SUI, animal or *in vitro* studies, non-injectable PRP applications (e.g., topical or intravesical administration), PRP used exclusively as an adjunct to surgical procedures, case reports, narrative reviews, editorials, letters to the editor, and conference abstracts lacking full-text availability.

### 2.3 Study selection and data extraction

Two independent reviewers (initials blinded) conducted title and abstract screening, followed by full-text review of potentially eligible articles. Discrepancies were resolved by consensus or consultation with a third reviewer. Data were extracted using a standardized form, including study characteristics (authors, year, country, and design), participant demographics, PRP preparation and administration protocols, comparator groups (if applicable), follow-up duration, and reported outcomes.

### 2.4 Outcomes of interest

The primary outcomes assessed in this study encompassed four key domains. First, symptom severity was evaluated using validated patient-reported instruments, including the Visual Analog Scale (VAS), the Global Response Assessment (GRA), and the Urogenital Distress Inventory–Short Form (UDI-6), each of which captures the subjective burden of stress urinary incontinence (SUI) from the patient’s perspective. Second, urodynamic parameters were analyzed, with specific attention to abdominal leak point pressure (ALPP), residual urine volume (RU), first sensation to void (FS), and functional profile length (FPL), which together provide objective measures of lower urinary tract function. Third, quality of life outcomes were determined using the Incontinence Impact Questionnaire–Short Form (IIQ-7) and the International Consultation on Incontinence Questionnaire–Short Form (ICIQ-SF), both widely accepted tools for assessing the psychosocial and functional impact of urinary incontinence. Lastly, safety outcomes were evaluated based on the incidence and severity of adverse events (AEs) following PRP administration, including both local and systemic complications as reported in the included studies.

### 2.5 Risk of bias assessment

Risk of bias was evaluated according to the study design. For randomized controlled trials (RCTs), the Cochrane Risk of Bias 2.0 (RoB 2) tool was employed, which assesses five key domains: randomization process, deviations from intended interventions, missing outcome data, measurement of the outcome, and selection of the reported result. For non-randomized before–after studies, the National Heart, Lung, and Blood Institute (NHLBI) Quality Assessment Tool for Before-After Studies With No Control Group was used, consisting of 14 criteria to evaluate internal validity, including clarity of the research question, specification of the study population, adequacy of follow-up, exposure and outcome measurement, and control of confounding variables.

Two reviewers independently assessed all studies. Any discrepancies in judgments were resolved by consensus or adjudication by a third reviewer. The risk of bias evaluation was used to inform the interpretation of pooled results and to guide the sensitivity analysis where appropriate.

### 2.6 Statistical analysis

Meta-analyses were performed using Review Manager (RevMan, version X.X, Cochrane Collaboration). For continuous variables, pooled mean differences (MD) with 95% confidence intervals (CI) were calculated. Heterogeneity was assessed using the *I*^2^ statistic, with values of 25, 50, and 75% representing low, moderate, and high heterogeneity, respectively. A random-effects model was used when substantial heterogeneity was present; otherwise, a fixed-effects model was applied. Sensitivity analyses and publication bias assessments were planned if ≥10 studies were available per outcome, though the limited number of included studies precluded formal funnel plot analysis.

## 3 Results

### 3.1 Search results and baseline characteristics

**Figure 1:**
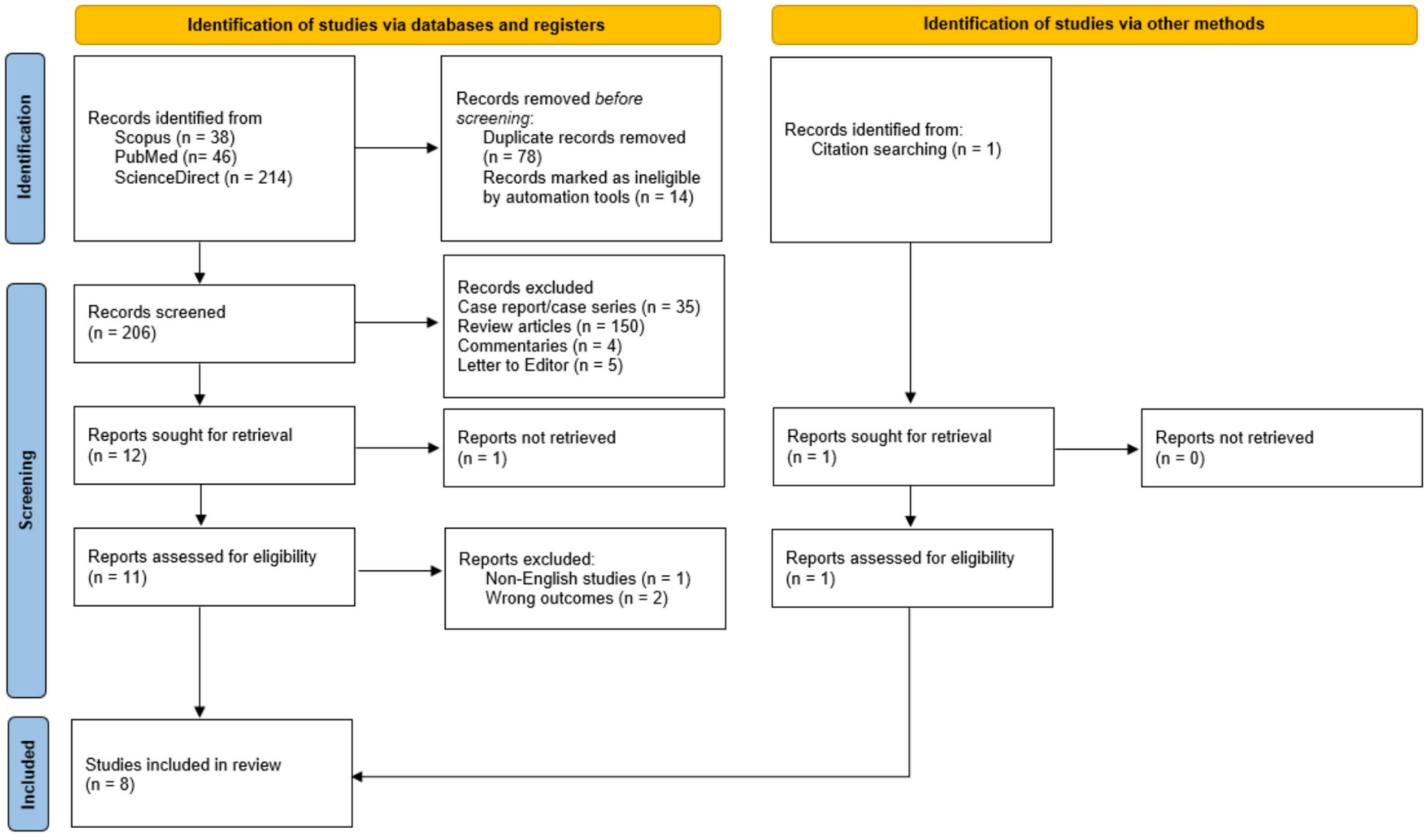
The results of screening articles are based on the PRISMA protocol.

Initial searches of three databases yielded a total of 298 records. After removing duplicates and ineligible records, 206 records were undergoing title-abstract screening. After independent screening, 12 records were selected for full-text review, resulting in 7 records being eligible for analysis. Subsequent citation searching was also conducted, resulting in the inclusion of 1 record. In total, this review analyses eight studies, consisting of three RCTs and five quasi-experimental studies across five different countries (Figure 1). The baseline characteristics of the included articles are available in Table 1.

**Table 1:** Baseline characteristics.

First author (year)	Countries	Sites	Study design	Total sample	Mean age	PRP delivery	PRP dose	Comparator
Azar Daneshpajooh et al. (2021) (8)	Iran	Kerman University of Medical Science	RCT	10 vs. 10	50.9 ± 2.76 (Exp); 45.7 ± 1.99 (Ctrl)	Cystourethroscopic injection at four sites along the mid-urethra	3 mL total	Midurethral sling
Fariba Behnia-Willison et al. (2020) (9)	Australia	Flinders Medical Centre, Adelaide	Before-after/ quasi-experimental study	62	55.98 ± 11.27	Transvaginal injection into the anterior lower one-third of the vagina and the periurethral area	NR	-
Ching-Hsiang Chiang et al. (2022) (10)	China	Hualien Tzu Chi Hospital	Before-after/ quasi-experimental study	26	61.7 ± 15.3	Periurethral injection at 5 sites (2, 5, 7, 10, 12 o’clock)	5 mL total (1 mL/site)	-
Yuan-Hong Jiang et al. (2021) (6)	China	Tzu Chi University, Hualien	Before-after/ quasi-experimental study	35	68.7 ± 12.0	Periurethral injection at 5 sites via perineal approach	5 mL total (1 mL/site)	-
Cheng-Yu Long et al. (2021) (11)	China	Kaohsiung Municipal Siaogang Hospital	Before-after/ quasi-experimental study	20	44.5 ± 9.1	Transvaginal injection at the mid-urethral area (1 cm below the meatus)	5 mL total (2 mL midline, 1.5 mL each side)	-
Apisith Saraluck et al. (2023) (12)	Thailand	Faculty of Medicine Ramathibodi Hospital, Mahidol University	RCT	29 vs. 31	51.76 ± 10.11 (Exp); 52.42 ± 9.30 (Ctrl)	Transvaginal injection at the mid-urethral area (1 cm below the meatus)	5 mL total (2 mL midline, 1.5 mL each side)	Pelvic floor muscle training only
Ahmed Samy Tahoon et al. (2022) (13)	Egypt	Sayed Galal hospital	Before-after/quasi-experimental study	20	48.65 ± 6.9	Injection into the anterior vaginal wall (site not specified)	NR	-
Leah Ashton et al. (2024) (14)	United States	Department of Urology, University of Iowa	RCT	25 vs. 25	47.67 ± 11.11 (Exp); 45.67 ± 10.37 (Ctrl)	Injection at 3 sites on the anterior vaginal wall (1 cm proximal to the meatus)	5 mL total (3 sites)	Placebo

Total sample is reported as intervention vs control when applicable. Single-group pre-post studies are indicated accordingly. Data are presented as mean ± standard deviation (SD). “Exp” indicates experimental/intervention group; “Ctrl” indicates control group. RCT: randomized controlled trial; NR = not reported.

### 3.2 Baseline characteristics and quality assessment of included studies

A total of eight studies were included, comprising three RCTs and five quasi-experimental (before-after) studies. The studies were conducted across six countries: Iran, Australia, China, Thailand, Egypt, and the United States. The pooled sample size consisted of 257 participants. The majority of participants were middle-aged to older adult females, with reported mean ages ranging from 44.5 to 68.7 years. The complete baseline characteristics are available in Table 1.

From quality assessment, all three RCTs were assessed as high quality, showing low risk of bias across at least five key domains. Among the quasi-experimental studies, four were rated as fair quality, and one study was assessed as having good methodological quality (see Table 2).

**Table 2:** Quality assessment.

NHLBI study quality assessment tools 2013					
Study	Behnia-Willison et al.	Chiang et al.	Jiang et al.	Long et al.	Tahoon et al.
1. Was the research question or objective in this paper clearly stated?	Yes	Yes	Yes	Yes	Yes
2. Was the study population clearly specified and defined?	Yes	Yes	Yes	Yes	Yes
3. Was the participation rate of eligible persons at least 50%?	Yes	Yes	Yes	Yes	Yes
4. Were all the subjects selected or recruited from the same or similar populations (including the same time period)?; Were inclusion and exclusion criteria for being in the study prespecified and applied uniformly to all participants?	Yes	Yes	Yes	Yes	Yes
5. Was a sample size justification, power description, or variance and effect estimates provided?	Yes	Yes	Yes	Yes	Yes
6. For the analyses in this paper, were the exposure(s) of interest measured prior to the outcome(s) being measured?	Yes	Yes	Yes	Yes	Yes
7. Was the timeframe sufficient so that one could reasonably expect to see an association between exposure and outcome if it existed?	Yes	Yes	Yes	Yes	Yes
8. For exposures that can vary in amount or level, did the study examine different levels of the exposure as related to the outcome (e.g., categories of exposure, or exposure measured as continuous variable)?	No	No	No	No	No
9. Were the exposure measures (independent variables) clearly defined, valid, reliable, and implemented consistently across all study participants?	No	Yes	Yes	Yes	No
10. Was the exposure(s) assessed more than once over time?	NA	NA	NA	NA	NA
11. Were the outcome measures (dependent variables) clearly defined, valid, reliable, and implemented consistently across all study participants?	Yes	Yes	Yes	Yes	Yes
12. Were the outcome assessors blinded to the exposure status of participants?	No	No	No	No	No
13. Was loss to follow-up after baseline 20% or less?	Yes	Yes	Yes	Yes	Yes
14. Were key potential confounding variables measured and adjusted statistically for their impact on the relationship between exposure(s) and outcome(s)?	No	No	Yes	No	No
Overall quality check	Fair	Fair	Good	Fair	Fair

Cochrane’s risk of bias 2.0			
Study	Daneshpajooh et al.	Saraluck et al.	Ashton et al.
1. Bias arising from the randomization process	Low-risk	Low-risk	Low-risk
2. Bias due to deviations from intended interventions	Low-risk	Low-risk	Low-risk
3. Bias due to missing outcome data	Low-risk	Low-risk	Low-risk
4. Bias in measurement of the outcome	Low-risk	Low-risk	Low-risk
5. Bias in the selection of the reported result	Low-risk	Low-risk	Low-risk
Overall quality check	Low-risk	Low-risk	Low-risk

NA: not applicable; NR: not reported.

### 3.3 Symptom severity and urodynamic outcomes

**Figure 2:**
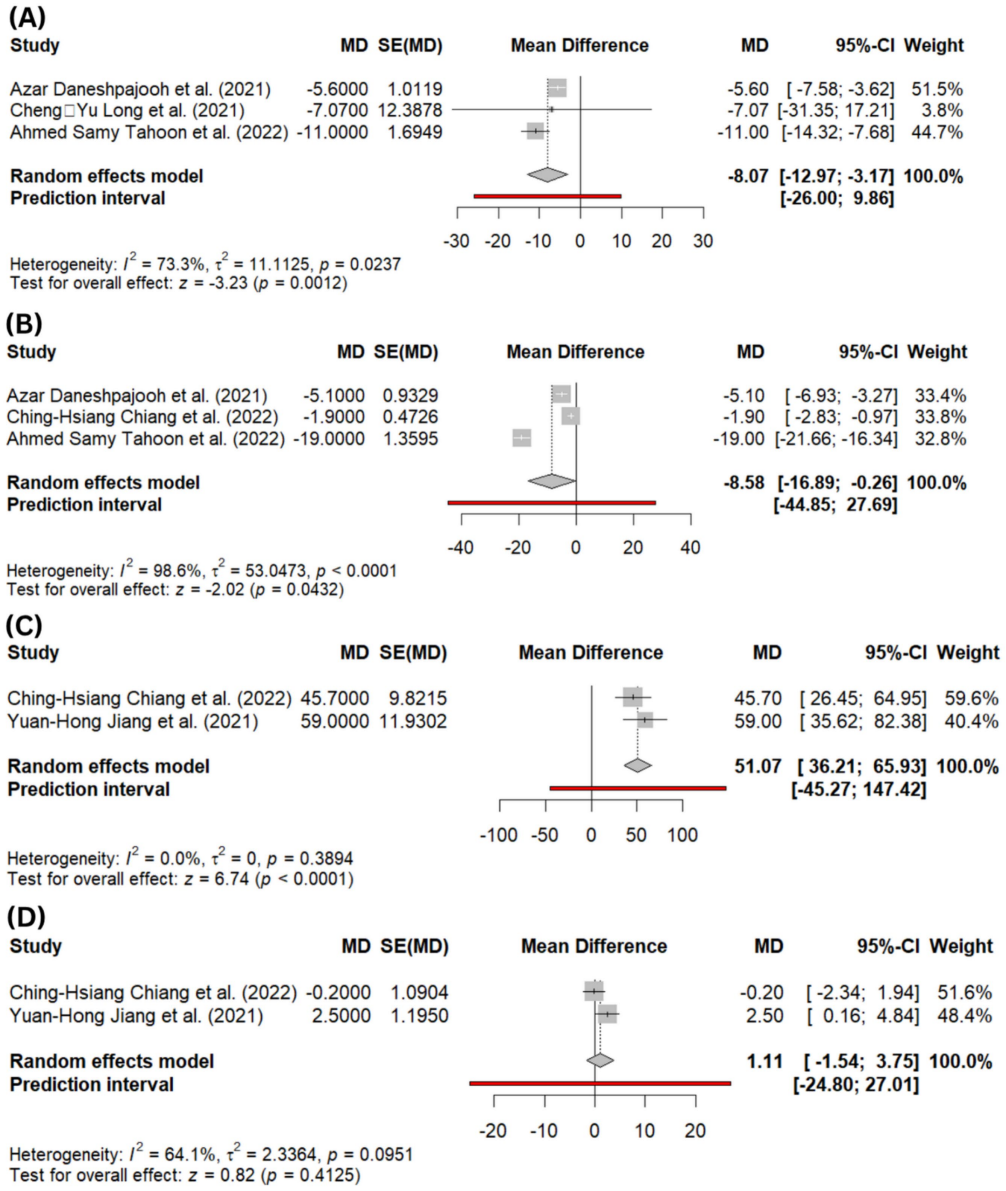
**(A)** Forest plot showing pooled mean difference (MD) in Urogenital Distress Inventory (UDI-6) scores at 1-month follow-up, **(B)** forest plot showing pooled MD in UDI-6 scores at 3-month follow-up, **(C)** forest plot of abdominal leak point pressure (ALPP) at 3-month follow-up, **(D)** forest plot of functional profile length (FPL) at 3-month follow-up.

**Figure 3:**
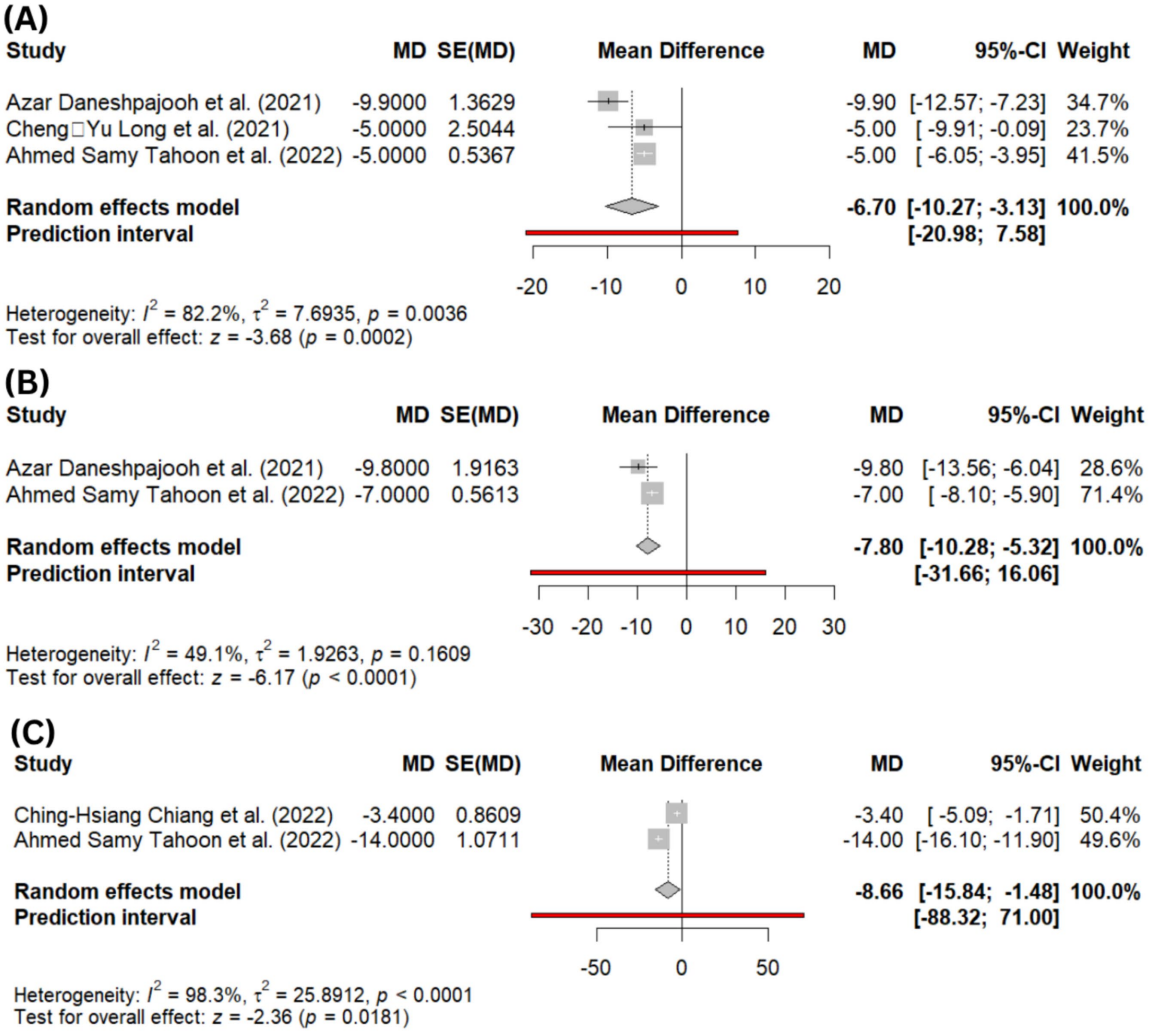
**(A)** Forest plot showing pooled mean difference (MD) in ICIQ-SF (International Consultation on Incontinence Questionnaire–Short Form) scores at 1-month follow-up, **(B)** forest plot showing pooled MD in ICIQ-SF scores at 3-month follow-up, and **(C)** forest plot of IIQ-7 (Incontinence Impact Questionnaire–Short Form) scores at 3-month follow-up.

Based on symptom severity changes, eight studies reported trends toward significant improvement during follow-up, ranging from post-operative to 12-month follow-up (6, 8–14). Two studies using GRA ≥ 2 showed success rates ranging from 50% - 60% (6, 10). Significant reductions in SUI VAS scores were reported at 3 months and 12 months (6, 10). Behnia-Willison et al. observed substantial improvement in SUI symptoms based on AFPQ scores at 3 and 6 months, while Saraluck et al. reported a significant reduction in 1-h pad weight in the PRP and pelvic floor muscle training (PFMT) intervention group compared to PFMT alone after 5 months (*p* = 0.02) (9, 12). Pooled quantitative analysis also showed a significant improvement of the Urogenital Distress Inventory (UDI-6) score questionnaire at 1-month follow-up (MD = −8.07; 95% CI: −12.97, −3.17; *I*^2^ = 73.3%; *p* = 0.001; Figure 2A) and 3-month follow-up (MD = −8.58; 95% CI: −16.89, −0.26; *I*^2^ = 98.6%; *p* = 0.04; Figure 2B).

Four studies reported urodynamic outcomes across multiple parameters (6, 10, 11, 13). Two studies demonstrated significant increases in residual urine (RU) and first sensation to void (FS) at 3- and 6-month follow-up (11, 13). Pooled analysis of abdominal leak point pressure (ALPP), defined as the intravesical pressure at which urine leakage occurs due to increased abdominal pressure in the absence of detrusor contraction, showed a marked improvement at 3 months (MD = 51.07; 95% CI: 36.21, 65.93; *p* < 0.0001; *I*^2^ = 0.0%; Figure 2C). However, the functional profile length (FPL), defined as the length of the urethra over which the pressure exceeds intravesical pressure, remained comparable to baseline, with no significant change at 3 months (MD = 1.11; 95% CI: −1.54, 3.75; *p* = 0.4; *I*^2^ = 64.1%; Figure 2D).

### 3.4 Quality of life improvement

Quality of life outcomes were assessed using two validated instruments: the Incontinence Impact Questionnaire–Short Form (IIQ-7) and the International Consultation on Incontinence Questionnaire–Short Form (ICIQ-SF). Pooled quantitative analysis showed the ICIQ-SF score showed significant improvement, with lower scores observed at 1-month (MD = −6.70; 95% CI: −10.27 to −3.13; *p* = 0.00002; *I*^2^ = 82.2%; Figure 3A) and 3-month follow-up (MD = −7.80; 95% CI: −10.28 to −5.32; *p* < 0.00001; *I*^2^ = 49.1%; Figure 3B). Similarly, the IIQ-7 score significantly decreased at 3 months (MD = −8.66; 95% CI: −15.84 to −1.48; *p* = 0.01; *I*^2^ = 98.3%; Figure 3C), reflecting enhanced quality of life associated with the use of PRP.

### 3.5 Safety outcomes

Across six studies reporting AEs, no life-threatening complications occurred (6, 8–10, 12, 14). Three studies reported that no AEs occurred in the PRP intervention (8, 9, 12). Chiang et al. noted one case of transient straining during urination, which resolved with self-catheterization (10). Jiang et al. reported mild hematuria and dysuria in 28.6% of patients, with all instances resolving conservatively; no urinary retention or infection was observed (6). Ashton et al. reported vaginal spotting and discomfort, with Clavien-Dindo Grade I events in 30.7% of the treatment group and 40.6% of the placebo group (14). Overall, all AEs observed were mild and self-limiting, supporting the safety profiles of PRP for SUI.

## 4 Discussion

This meta-analysis synthesizes evidence from eight clinical studies, including randomized controlled trials and quasi-experimental designs, to evaluate the potential role of PRP in the management of SUI in women. Overall, pooled analyses suggested improvements in symptom severity and patient-reported quality-of-life measures, particularly during the early post-treatment period. However, these findings should be interpreted cautiously in light of the limited sample sizes and methodological heterogeneity of the included studies.

Platelet-rich plasma is an autologous blood-derived concentrate enriched with platelets and a broad spectrum of bioactive growth factors, including vascular endothelial growth factor (VEGF), transforming growth factor-beta (TGF-*β*), platelet-derived growth factor (PDGF), insulin-like growth factor (IGF), and basic fibroblast growth factor (bFGF) (4, 15, 16). Upon activation, platelets release these mediators, which act synergistically to promote angiogenesis, extracellular matrix remodeling, myogenesis, and neural repair, processes that are essential for tissue regeneration and functional recovery (15, 17, 18). In the context of SUI, PRP is hypothesized to exert its therapeutic effects by enhancing the structural and functional integrity of the urethral sphincter complex and periurethral supportive tissues. Experimental and translational studies suggest that PDGF and TGF-β facilitate fibroblast recruitment and collagen synthesis, thereby improving connective tissue strength and sphincter support, while VEGF-driven neovascularization enhances local perfusion and metabolic support to regenerating tissues (17–19). In addition, emerging evidence indicates that PRP may stimulate myogenic differentiation and neuromuscular regeneration, potentially improving sphincter contractility and urethral closure competence (6, 19–21). Nevertheless, while these mechanistic pathways are biologically plausible and supported by preclinical and early clinical data, the extent to which PRP-induced regeneration translates into durable and clinically meaningful improvement in SUI remains to be conclusively established through adequately powered, long-term randomized controlled trials.

Regarding urodynamic outcomes, significant improvements in ALPP were observed in pooled analyses. However, it is important to note that this finding was primarily driven by only two small quasi-experimental studies, limiting the robustness and generalizability of the observed effect. The absence of consistent ALPP reporting across larger randomized trials and the short follow-up durations further constrain the interpretation of this outcome. Consequently, while ALPP may represent a sensitive marker of early sphincteric functional change, the current evidence is insufficient to support definitive conclusions regarding its responsiveness to PRP therapy In contrast, FPL did not demonstrate a significant change following PRP treatment. As FPL reflects a structural aspect of urethral function, it may be inherently less responsive to short-term regenerative interventions. Alternatively, changes in FPL may require longer observation periods, repeated PRP administrations, or may not be directly modulated by PRP-related biological mechanisms. These findings underscore the need to distinguish between functional and structural urodynamic parameters when interpreting treatment effects.

Some inconsistencies were also evident in subjective outcome measures. Specifically, two studies reported non-significant improvements in the GRA following PRP injection (6, 10). Given that GRA relies heavily on patient perception, it is susceptible to recall bias, placebo effects, and interindividual variability. Differences in assessment timing and the lack of standardized baseline-adjusted analyses may have further limited the sensitivity of this measure to detect modest or early improvements.

Although this meta-analysis integrates multiple outcome domains, including symptom scores, urodynamic parameters, and validated quality-of-life measures, to provide a comprehensive quantitative synthesis, the findings should be interpreted as exploratory rather than confirmatory. The small number of included studies, modest sample sizes, and substantial heterogeneity in PRP preparation protocols, injection techniques, anatomical injection sites, and follow-up durations reduce the certainty of pooled effect estimates. Collectively, these limitations highlight the need for well-designed, adequately powered randomized controlled trials with standardized PRP protocols and harmonized outcome measures to more reliably define the efficacy, safety, and durability of PRP therapy in female SUI.

Future studies should incorporate a minimum set of standardized reporting and procedural elements to enhance reproducibility and comparability across trials. These should include detailed characterization of PRP preparation, such as baseline platelet count, centrifugation technique and parameters, final platelet concentration, and activation status. Procedural aspects, including injected volume per session, injection technique (transvaginal, transurethral, periurethral, or cystourethroscopic), anatomical injection site and depth, as well as the number and interval of treatment sessions, should be clearly defined and justified. Furthermore, the adoption of a core outcome set encompassing validated symptom severity scores, objective urodynamic parameters, quality-of-life measures, and prespecified short- and long-term follow-up time points is essential. Establishing such standardized protocols would facilitate meaningful cross-study comparisons and support robust evaluation of the long-term clinical role of PRP in the management of female SUI.

## 5 Conclusion

PRP appearspromising in alleviating symptoms, improving urodynamic outcomes, and enhancing quality of life in women with SUI. The absence of serious adverse events across studies supports the overall safety and clinical tolerability. Future research should focus on standardized dosing protocols to optimize clinical application.

## Data Availability

The original contributions presented in the study are included in the article/supplementary material, further inquiries can be directed to the corresponding author.
